# Patient-Perceived Autonomy and Long-Acting Reversible Contraceptive Use: A Qualitative Assessment in a Midwestern, University Community

**DOI:** 10.1089/biores.2017.0037

**Published:** 2018-03-01

**Authors:** Carley Zeal, Jenny A. Higgins, Shaunna R. Newton

**Affiliations:** ^1^ Division of Family Planning, Department of Obstetrics and Gynecology, Washington University School of Medicine in St. Louis, St. Louis, Missouri.; ^2^ Department of Obstetrics and Gynecology, Gender and Women's Studies, University of Wisconsin-Madison, Madison, Wisconsin.; ^3^ School of Public Health, University of Maryland College Park, College Park, Maryland.

**Keywords:** autonomy, contraceptive, counsel, long-acting reversible contraceptive

## Abstract

Long-acting reversible contraceptives (LARCs) are the most effective contraceptives and are first-line recommendations for most women. However, young women use these methods at relatively low rates. Given concern with contraceptive coercion, an underexamined factor contributing to LARC attitudes is women's perceived reproductive and bodily autonomy in regard to LARC. We conducted focus group discussions and interviews regarding LARC perceptions and knowledge with 50 women between the ages of 18 and 29. We used a modified grounded theory approach to analyze young women's impressions of autonomy in relation to contraceptives more generally and LARC more specifically, both among ever-users and never-users. Four themes emerged regarding women's perceived autonomy with LARC. Control over pregnancy, active participation versus external agent, control over bleeding patterns, and autonomy in the provider/patient relationship. Within most themes, women made both positive and negative associations between perceived autonomy and LARC. The provider/patient relationship was a modifier of other themes, in that cooperative relationships may overshadow other perceived reductions in autonomy, and more unbalanced relationships may heighten perceived reductions in autonomy. Ever-users were more likely to report increased autonomy with LARC use, whereas never-users were more likely to express concerns about loss of autonomy with LARC. This study suggests that perceived autonomy may influence women's perceptions of LARC as well as their uptake of these contraceptive methods, with several factors both positively and negatively related to women's perceived autonomy. We encourage the integration of these findings into patient-centered counseling as well as educational materials for LARC.

## Introduction

Long-acting reversible contraceptives (LARCs) are widely accepted as the most effective forms of birth control.^1^ They are widely becoming the most recommended form of contraception by providers, for multiple patient populations.^2^ However, despite intrauterine devices' (IUDs) and implants' strong efficacy and acceptability, there is still a relatively low uptake of these devices by young adults in the United States. In 2012, IUDs and implants were used by only 15% of contracepting women between the ages of 18 and 29^3^—the age group with the greatest burden of unintended pregnancy.^4^

Researchers have documented a variety of factors that may affect women's willingness to try LARC methods. Facilitators have included reliability and efficacy,^5–7^ duration of action,^7^ positive perceptions from family and friends,^5,8^ recommendation from a provider,^8^ and low-maintenance nature of the methods.^6^ Factors negatively associated with LARC use have included fear of side effects,^6,9^ apprehension about pain for insertion,^7,9,10^ and communication of negative experiences.^11^ Some factors may act as both barriers and facilitators, depending on the person. Taken cumulatively, these studies suggest that women's decisions to use or not use a LARC are complex considerations.

One important but largely underexplored contributor to contraceptive choices is perceived autonomy, or rather, patients' values regarding reproductive and bodily autonomy. This question of a contribution of autonomy to contraceptive decision-making, specifically perceptions of LARC, stems from a long history of questionable practices or contraceptive coercion. It is well documented that developers of some of the first contraceptives used racist and eugenicist arguments, and a history of forced sterilization in the United States cannot be ignored.^12^ Concerns for biased contraceptive counseling remain, particularly among low-income patients of color.^13^ As recently as the 1980s, the contraceptive implant, Norplant, was specifically advertised to women of minority populations and low socioeconomic status, and in the mid-90s, several states proposed legislation providing financial incentives for women receiving public assistance if they had a contraceptive implant placed.^12,14^ With such biased provision of contraceptive methods, it is more than understandable that women desire autonomy in their contraceptive decision-making, even more so than in other medical decisions.^15^ However, despite the data exploring the influence of contraceptive counseling on women's decision-making,^1^ we have little understanding of how reproductive and bodily autonomy influences women's perceptions of LARC methods.

This study aims to use qualitative methods to explore this interaction in young adult women by identifying themes related to both women's sense of reproductive and bodily autonomy and use of a LARC, as a subanalysis of data of these women's general knowledge and perceptions of LARC.

## Materials and Methods

### Study design

Data for this analysis derive from a larger qualitative study^2^ of IUD and implant use among 18–29-year-old women who had used reversible contraception of any kind in (small Midwestern city, blinded for review purposes) a semiurban area of ∼500,000 inhabitants and home to the University of Wisconsin. Approximately 13% of residents live below the federal poverty level (compared with 15% nationally), and 19% of residents are people of color (compared with 23% nationally).^16^ We selected the 18–29-year-old age group given their disproportionate burden of unintended pregnancy^4^ and their comparatively low likelihood of using LARC.^17^ We also selected a qualitative approach for the study given that qualitative research methods are essential for exploring understudied topics, generating hypotheses (vs. showing causation), and answering questions of why, how, and under what circumstances versus how many.^18^

In phase 1 of the larger study, investigators conducted focus groups with women who had any history of contraceptive use. To recruit participants, study team members posted and distributed flyers in university buildings, public libraries, Planned Parenthood clinics, university health services, other health clinics (e.g., federally qualified health centers), bus shelters, and Job Corps offices. In addition, recruitment e-mails were circulated to university groups; public health departments; representatives of the Special Supplemental Nutrition Program for Women, Infants, and Children (WIC); and other pertinent health and social organizations. We also posted information about the study in the community volunteer and “etc.” jobs sections of Craigslist, and in a free local weekly newspaper. Some participants were referred by friends or family members who qualified for the study.

Focus groups were designed to explore young adult women's LARC-related knowledge and attitudes, as well as various factors associated with LARC acceptability and access.^19^ To more deeply explore personal experiences of women, we conducted 12 one-on-one interviews (phase 2). Given the small number of LARC users, and the generalized exploratory nature of the study for LARC perceptions, no differentiations were made in this data set between users of IUDs versus implants. Focus group questions included the following categories: contraception in general (e.g., “What kinds of things are important to women when they choose a birth control method?”) and IUDs and implants more specifically (e.g., “What are positive and negative things you have heard about IUDs,” “Do you think some women are more likely to use IUDs than other women, Why,” “How easy or difficult might it be for someone to get an IUD or implant?”).

To ensure socioeconomic diversity among participants, we designed a stratified sampling frame: one-third of focus groups and interviews were with current university students, and two-thirds were with women from the community currently receiving at least one form of public assistance. Other inclusion criteria included being between 18 and 29 years of age and having any history of contraceptive use, excluding women who had not used reversible contraception. Although race and ethnicity were not part of our sampling frame, we strove for racial and ethnic diversity among both university and community respondents.

Before any data collection, the University of Wisconsin Institutional Review Board reviewed and waived the study design and instruments. It is important to note that this analysis represents a subanalysis of this data set.

### Data collection

Please see elsewhere for a more detailed description of our data collection and recruitment.^2^ To briefly summarize, data collection took place between January and June of 2014. Focus groups were designed to explore young adult women's LARC-related knowledge and attitudes, as well as various factors (e.g., medical, relational, sexual) associated with LARC acceptability. We selected focus groups because of their utility in measuring social norms, expectations, and values (vs. individual experiences). For example, we asked focus group participants what they knew, if anything, about IUDs and implants, and about both positive and negative aspects of these methods. We also wanted to more deeply explore personal experiences of women who had ever used a LARC method. Therefore, we conducted one-on-one interviews with former or current LARC method users. Interviewees answered questions about their own decisions to get an IUD or implant and about their experiences with their LARC method.

Focus groups contained 4–10 participants and lasted between 1.5 and 2.5 h. Interviews lasted between 25 and 55 min. At the conclusion of the focus group or interview, university participants received $20 gift cards and community participants received $30 gift cards, due to differences in resources needed for participation. All focus groups and interviews were audio-recorded and then transcribed by either a study team member or an independent transcription service. Focus groups and interviews were not written to specifically incite participants' perceptions of autonomy.

### Data analysis

We used an inductive, modified grounded theory approach in analyzing the data, meaning that we drew on preexisting themes from the literature and research questions as well as themes arising from the data. We first organized interview and focus group transcripts from the collected data into coding reports comprising a wide range of codes relating to our research question. The second author generated the initial codes halfway through the collection with two graduate student assistants and then winnowed the list. Trained research team members applied codes to relevant blocks of text in each transcript. Two coders independently coded each transcript and then met to discuss the codes. Once 100% agreement was reached, one coder entered all codes from an individual transcript into Atlas.ti.

Since “autonomy” was not an initial focus of the data collection team, the original code list did not include a code that specifically pertained to participants' perceptions of autonomy and LARC. Thus, for the current analyses, the three authors of this article analyzed a wider range of codes with salience for autonomy. The first and second author reviewed reports for each of these codes, independently identified themes pertaining to perceived autonomy, and then met to refine these themes into a master list of seven. The third author then pulled data pertaining to these themes, creating memos. The first and third author then reviewed these memos and used a tallying system to assess differences within each theme across those who had used or were currently using a LARC, and those who had not (ever-users and never-users). Finally, to make the themes more manageable for publication, the first and second authors condensed and created a final list of four themes more directly related to LARC.

Quotations from focus groups are not fully comparable to quotations from interviews as units of analysis, given the inherently different dynamics of these two data collection mechanisms. However, given the exploratory nature of our study, as well as the fact that focus group participants did share both personal and anecdotal stories (as opposed to merely attitudes and larger social norms), we mix both interviewee and focus group data in our presentation of results.

## Results

With regard to the study population, there were a total of 50 participants, of those 16 had used a LARC or were currently using a LARC. We conducted six focus groups with 40 women who had any history of contraceptive use; of those, 16 had used a LARC. Please see Table 1 for demographic data of the study population.

**Table 1.** **Overview of Study Participants**

	FG participants (6 groups, *N* = 40)	Interview participants (*N* = 12)	All^a^ (*N* = 50)
University students	19	4	23
Community residents			
receiving public assistance	21	8	27
Race/ethnicity^b^			
White	22	10	32
Black	5	1	5
Latina	6	—	6
Asian	3	—	3
Native American	2	—	2
Biracial	3	1	3
Highest level of education			
High school	2	—	2
Some college	24	6	29
College	12	4	15
Any history of LARC use	8	10	16

^a^ Two focus group participants also participated in interviews.

^b^ Two participants selected more than one race.

FG, focus group; LARC, long-acting reversible contraceptive.

With regard to comparisons between ever-users and never-users, ever-users of LARC frequently reported increased reproductive and bodily autonomy related to their use of their IUD or implant. In addition, never-users were more likely to express negative associations between aspects of LARC use and perceived autonomy.

Below we present four themes that related to participants' perceived autonomy in relation to LARC methods. We present the first three themes—control over pregnancy, active participation versus external agent, and control over bleeding patterns in order of how frequently they appeared in the data. We present the patient/provider theme last, given its role as a modifier of the other three themes.

### Control over pregnancy

With regard to women's control over pregnancy, the data revealed a dichotomy between women who sensed increased control with LARC's efficacy and ease of use on one hand, and women who felt LARC could reduce their autonomy by reducing their reproductive flexibility. These sentiments were mixed among women, despite their history of LARC use or nonuse.

Many women expressed that LARC methods offered peace of mind by way of their low maintenance and ease of use, particularly compared to shorter acting methods. Many women described a kind of freedom from having to maintain a method, while maintaining the security of efficacy. One community focus group participant who had used a LARC said:

With other methods that I've used, you know, there's still a possibility if you don't use it correctly… But [the IUD] pretty much seems like unless you know that it's falling out, you're 100% guaranteed to not have to think about it.—Amy

Women reported that enhanced control over reproduction provided enhanced freedom to control other aspects of their lives. For example, the following quotations were reported by community interviewees who had used LARC:

I feel more in control of my future. […] It's just easier and it doesn't stress me out that I could get pregnant.—Hope

I could definitely see where a woman who wants something a little bit more permanent that she doesn't have to worry about every day because she wants to get her career out of the way like before she has kids. […] She can control, you know, kind of when she wants to have her kids.—Penny

Other women reported that the efficacy of LARCs may reduce their sense of control over their reproduction. Women described a spectrum of willingness to conceive, and those women who were ambivalent or unclear about their own desire for a pregnancy could dislike the security against pregnancy afforded by LARC. These women sensed decreased control due to the efficacy of LARC. For example, here is one exchange with a community focus group participant who was a non-LARC user:

Facilitator: So, you are saying you don't have a lot of control over the IUD's effects on your fertility?

Ivy: Right … [the IUD] takes the element of surprise out of when we would have our next kid, which I kind of want.—Ivy

Finally, in regard to efficacy, some women reported that the long-term aspect of LARCs diminished some sense of control over their future fertility. Despite reported understanding of reversibility of the methods, some women reported a decreased sense of control over return of fertility. Although this illustrates some misunderstanding regarding the method's reversibility or skepticism regarding the information provided, it does seem to influence women's sense of autonomy with regard to LARCs. One university interviewee who was a LARC user said:

Hope: When people think about long-term birth control, they think this is a five-year commitment. Like, I'm locked into this birth control for five years, whereas with the pill, you can change it every month.

Facilitator: So you, even if you don't like it, you have that control?

Hope: Yeah.—Hope

### Active participation versus an external agent

Women described the importance of a sense of physical control over one's body and reproduction, and contraceptives served as an extension of such control. However, contraceptive methods could also be perceived as external agents acting on women's bodies, controlling their reproduction. While LARCs were seen as effective at controlling reproduction, they were also perceived as foreign bodies, removing an active role of the woman in her contraceptive method, and requiring a provider for placement and removal. However, LARCs were more desirable for some women, who perceived hormones in shorter acting methods as an external agent, affording options with less or no hormones.

For example, some women took comfort in having control over the physical act of taking a pill or otherwise actively participating in behaviors related to contraception. A community focus group participant who did not use LARC expressed this sentiment:

Coming back to the control thing, … but there's just something mental about, you know, you're taking the pill, you know you swallowed it.—Ivy

Similarly, women described a discomfort or loss of control over the idea of a foreign object in their body. One university interviewee who had used a LARC method said:

I think that […] people are uncomfortable having something constantly in them that's they feel like they're not in control of it.—Hope

In addition, some women described a hesitancy to use a LARC due to their decreased sense of control of reproductive decision-making, because they require another person to insert or remove. This phenomenon is illustrated by a non-LARC user from the community who participated in a focus group:

Yeah, I'm fine putting something else in me, myself, …but like having a procedure and somebody else sticking something in my uterus was just kind of weird. I was like, ‘no thanks.’ If I can't like, you know, deal with it myself I don't really want anybody messing with it.—Candice

Autonomy was particularly threatened when women encountered providers who were reluctant to remove their LARC device. Some providers prefer a trial of time with treatment of side effects before removal of a LARC, due to the expense of the device and the providers' perceived benefits over time. This created a power struggle between the woman and their provider, reducing their perceived control. A community interviewee who had used a LARC method said:

Getting [my IUD] taken out was a little bit frustrating. As far as I knew, I couldn't take [my IUD] out myself. And so having to argue back and forth with [a provider]…Because if you have something in your body that you can't take out, it's kind of a strange feeling.—Katie

While women reported desire to actively participate in their contraceptive method and direct control over starting and stopping the method, they also reported that exogenous hormones in methods were perceived as inherently bad, and controlling women's bodies. LARCs could be seen as more desirable or affording more autonomy, providing options with fewer or no systemic hormones. One community interviewee who had used a LARC method reported the lack of systemic hormones as an advantage:

And then another [positive] thing that my doctor told me is there is no hormones that go throughout your blood with the Mirena. There's just hormones that stay in the uterus.—Anna

Similarly, a university focus group participant who had never used a LARC method said:

[I don't like] just having hormones regulate your body and do different things to your body, whether you want it or not it's still doing things… And for me and for my friends that makes us really nervous at least having something man-made in there, controlling our body. We might have the illusion of control, but really it's something else doing something to our bodies.—Hayley

In sum, a dichotomy arose: While some women perceived LARCs as negative external agents, with providers having too much control over starting and stopping these methods, other women perceived exogenous hormones in other methods as the negative external agents controlling their bodies.

### Control over bleeding pattern

Some women reported that certain LARC methods afforded increased autonomy by allowing them to control their menstrual bleeding with decreased bleeding or no menstrual cycles. This sentiment was expressed specifically in regard to the levonorgestrel IUD. For example, one university interviewee who had used a LARC method reported:

I would say I like not getting my period … I will really like that.—Cameron

A community interviewee who had used a LARC method added to this sentiment by saying:

 … [I] don't get periods very often, hardly ever. That's really nice [laughs]. —Jessica

However, other women found either irregular bleeding or no bleeding disconcerting, because it did not allow for reassurance of contraceptive efficacy and thereby decreased their sense of control over their fertility. For example, one community interviewee who had used a LARC method said:

[My provider] was like, ‘but you're probably not going to get your period anymore; you're just going to get a little bit of spotting.’ And I was like, ‘see, I kind of like getting my period’ at the same time, because it lets me know that I'm not pregnant. …it's really a control thing—I know exactly when it's coming and even though it sucks at least I know like it's there and I have control over it.—Jessica

In sum, some women reported increased control over their menstrual cycles with a LARC, which increased their autonomy. Others felt decreased autonomy as a result of these agents altering their menstrual cycles, desiring a method more likely to result in cycles that more closely resemble their natural cycles or ones that feel more normal to them.

### Provider/patient relationship

Women described a direct association between the quality of the provider/patient relationship and their sense of contraceptive autonomy. This theme was particularly important because it influenced all the other stated themes. For instance, particularly if she expressed a mistrust of doctors and/or a history of difficult experiences as a contraceptive client, a woman could associate LARC with decreased autonomy. However, if she reported a trusting relationship with her provider, she was less likely to perceive provider influence as a threat to autonomy and more likely to perceive the provider as a trusted source of information and care.

Some women reported that placing LARCs and potential resistance to LARC removal were based on the providers' personal agendas, reducing women's control over contraceptive choices. One community interviewee who was a LARC user expressed:

I don't know if it makes [providers] look bad if you have an IUD removed and they're the one who placed it, or I don't know if [providers] have some stat chart somewhere, like a contest board in the breakroom.—Katie

Another university focus group participant who had never used a LARC method said:

I feel like [LARC insertion] brings to mind these visions of forced sterilization like after an abortion or after a birth, just like tying the tubes, and again like with the fear that goes along with long-term and the risks of maybe messing up your fertility.—Meredith

A community focus group participant who had used a LARC method stated:

It seems like young African-American women are more pressured [to use IUDs and implants], from my point of view, just from different things I've heard of or different experiences. …Although it seems like most adolescents these days, but I think more so it seems like African-American ones are being pressured more.—Amanda

Similarly, women reported a feeling of judgment from some providers in regard to their fitness to make reproductive choices. When women reported pressure for certain methods from an untrusted source, they sometimes sensed this was a reflection of the providers' impression of their fitness to choose the appropriate method. This sense of judgment made the LARCs seem like an agent of an outside agenda, reducing their autonomy in reproduction. Illustrating this view, a community interviewee who was a LARC user reported:

So I feel like you're maybe trying to tell me something, that you don't think that I'm responsible enough or that you don't think that I tried hard enough.—Jessica

One participant illustrated the sentiment well that women may feel more or less autonomy or more or less likely to choose a recommended method based on their relationship with their provider. This community focus group participant who had used a LARC method said:

I think it matters, too, if you had a doctor that you didn't feel like you had a good relationship with. Then I probably would be more like, ‘well, nah,’ because they're not going to listen to me. Versus if I felt like I had a pretty good relationship with my doctor. I think that makes a big difference, too.—Amanda

In sum, women reported increased autonomy in relation to contraceptive decision-making when working in a cooperative relationship with their provider, and decreased autonomy if that relationship was dysfunctional.

## Discussion

Few studies have explored the contribution of patient-perceived autonomy in relation to perceptions of LARC. However, this question has become particularly salient within the context of the reproductive justice movement and growing concern for reproductive coercion. In this study, we found not only that patient-perceived autonomy may influence perceptions of LARC but that the provider/patient relationship may play an important role in perceived autonomy as well. We also documented several other factors contributing to patient-perceived autonomy and LARC that varied notably across individuals.

First, we found that ever-users of LARC frequently reported increased control over pregnancy, their bodies, and their lives as a result of their IUDs or implants. This finding suggests that perceived reproductive and bodily autonomy may contribute to LARC use. Another qualitative study reported that participants expressed reproductive autonomy as a facilitator to LARC uptake.^8^ However, another qualitative study identified lack of control over IUDs as a theme in exploring factors related to nonuse of LARCs.^10^ Along those lines, some never-users in this study did express concerns about not having control over the insertion or removal process. However, among these never-users, LARC avoidance pertained more to lack of knowledge and understanding of these methods compared to concerns about autonomy. In other words, we found perceived autonomy to be more of a positive aspect of LARC use than a deterrent. Our findings reinforce that both positive and negative perceptions of autonomy arise with respect to LARC, but that it appears that a lack of understanding of the methods may contribute to negative perceived autonomy.

In terms of the key control-related themes that arose during analysis—that is, control over pregnancy, active participation versus an external agent, and control over bleeding patterns—women in our study reported both positive and negative associations with perceived autonomy. Some of women's responses appeared to depend on whether they perceived a LARC as a choice of their own versus an agent potentially acting on them. For example, some women feel liberated choosing a method that does not require daily input, while others report lack of control with a device placed inside them and removed by another person. These findings serve as yet another reminder that patients have individual preferences and responses to contraceptive methods, and the same contraceptive factor that will appeal to one woman might detract another. Along these lines, contraceptive counseling efforts should focus on individual patient needs and desires.^20^ However, our findings may nonetheless provide some cohesive suggestions for LARC counseling and education. For example, counselors and educators could highlight specific factors associated with increased autonomy, such as reversibility. We may also illustrate women's stories of increasing their control with regard to contraceptive decision-making regarding LARC.

Women in this study reported increased control over their contraceptive method and lives when they had a cooperative relationship with their provider. Unfortunately, women in this study more commonly voiced concerns than assurances regarding providers' LARC recommendations or removal practices. In keeping with other research, a number of women felt their providers were recommending a LARC or were reluctant to remove a LARC for reasons counter to the patient's needs.^21,22^ In this way, women could have a decreased sense of control in light of LARC. However, this relationship was reversed in a cooperative relationship. Recent research in contraceptive counseling has emphasized the importance of facilitating women identifying their own family planning needs, including an understanding of the social contexts within which these needs are prioritized.^23,24^ Studies also suggest that there are varying degrees of provider contribution to contraceptive decision-making, and this contribution may take place in regard to information gathering.^20,24^ It may be valuable to explore these patient-centered counseling methods within the context of patient-perceived autonomy in contraceptive decision-making, as well as reduce the sense of loss of control in the adjustment and reassessment phases of decision-making.

Findings should be considered in terms of study limitations. For example, the initial study was not designed to specifically explore women's perceived autonomy with regard to LARC. There may be inherent researcher bias: our exploration may overemphasize the importance of perceived autonomy with regard to contraceptive decision-making. However, given that we did not directly elicit participants' perceptions of their autonomy, our process allowed for participants to naturally express their opinions, without biased or leading questions. In addition, our small study population was relatively homogenous in terms of socioeconomic factors, including a large proportion of white participants with at least a high school diploma. Preferences for contraceptives as well as perceptions of contraceptive counseling relationships may vary based on some of this demographic information. Given that women of color have been bearers of reproductive injustices over time, it is likely that a larger sample of women of color would have resulted in more vehement concerns about threats to autonomy.^14^ We did observe some of this variation between community and university representatives in this study. However, results were similar to previous studies in regard to positive and negative predictors of LARC use. For example, women in this study reported the low-maintenance nature of LARCs as a positive aspect, similar to data from Dabrow, Kavanaugh, Glasier, and Weston.^6–8,22^ This suggests at least some degree of reliability and validity in our data.

In conclusion, this study suggests that perceived autonomy may influence women's perceptions of LARC as well as their uptake of these contraceptive methods, with several factors both positively and negatively related to women's perceived autonomy. These findings are important within the historical context of reproductive coercion and other studies suggesting the importance of autonomy in contraceptive decision-making.^12,15^ Results suggest that the provider/patient interaction may influence women's perceived autonomy, even acting as a modifier of other factors influencing their perceived autonomy. We encourage the integration of these findings into patient-centered counseling as well as educational materials for LARC. Integration of this understanding into educational material for LARCs for providers and patients may improve patient-centered counseling to assist women in choosing a contraceptive that aligns with their contraceptive needs, lifestyle, and improves their reproductive autonomy.

## Abbreviations Used

LARC: long-acting reversible contraceptive
IUD: intrauterine device
